# Whole-body diffusion-weighted magnetic resonance imaging versus FDG-PET/CT for initial lymphoma staging: systematic review on diagnostic test accuracy studies: Ressonância magnética de corpo total com difusão comparada à FDG-PET/CT no estadiamento inicial do linfoma: revisão sistemática de estudos de acurácia diagnóstica

**DOI:** 10.1590/1516-3180.2014.8312810

**Published:** 2015-03-17

**Authors:** Rodrigo Regacini, Andrea Puchnick, David Carlos Shigueoka, Wagner Iared, Henrique Manoel Lederman

**Affiliations:** I MD, MSc. Radiologist, Discipline of Pediatric Radiology, Department of Diagnostic Imaging, Universidade Federal de São Paulo-Escola Paulista de Medicina (Unifesp-EPM), São Paulo, Brazil.; II BSc. Professor and Coordinator of Educational and Research Support, Department of Diagnostic Imaging, Universidade Federal de São Paulo-Escola Paulista de Medicina (Unifesp-EPM), São Paulo, Brazil.; III MD, PhD. Adjunct Professor, Department of Diagnostic Imaging, Universidade Federal de São Paulo-Escola Paulista de Medicina (Unifesp-EPM), São Paulo, Brazil.; IV MD, PhD. Assistant Research Radiologist, Department of Diagnostic Imaging, Universidade Federal de São Paulo-Escola Paulista de Medicina (Unifesp-EPM), São Paulo, Brazil; V MD, PhD. Full Professor and Head of the Discipline of Pediatric Radiology, Department of Diagnostic Imaging, Universidade Federal de São Paulo-Escola Paulista de Medicina (Unifesp-EPM), São Paulo, Brazil.

**Keywords:** Magnetic resonance imaging, Diffusion magnetic resonance imaging, Whole body imaging, Lymphoma, Positron-emission tomography

## Abstract

**CONTEXT AND OBJECTIVE:**

Positron emission tomography with [18]F-fluoro-2-deoxyglucose (FDG-PET/CT) has been advocated as the method of choice for lymphoma staging, since it enables whole-body analysis with high sensitivity for detection of affected areas and because it combines capacities for anatomical and functional assessment. With technological advances, magnetic resonance imaging (MRI) has emerged as an alternative to FDG-PET/CT. This systematic review with meta-analysis aimed to compare whole-body diffusion-weighted MRI (WB-MRI) with FDG-PET/CT for lymphoma staging.

**DESIGN AND SETTING:**

Systematic review on diagnostic test accuracy studies conducted at a public university.

**METHODS:**

The Medline, Scopus, Embase and Lilacs databases were searched for studies published up to September 2013 that compared WB-MRI and FDG-PET/CT for lymphoma staging. The reference lists of included studies were checked for any relevant additional citations.

**RESULTS:**

Six studies that evaluated the initial lymphoma staging in 116 patients were included. WB-MRI and FDG-PET/CT agreed in 90.5% of the cases (κ = 0.871; P < 0.0001). In most of the studies, when there was disagreement between the methods, WB-MRI overstaged in relation to FDG-PET/CT. The sensitivity of WB-MRI and FDG-PET/CT, in comparison with the clinical-radiological standard, ranged from 59 to 100% and from 63 to 100% respectively.

**CONCLUSION:**

WB-MRI is a highly sensitive method for initial lymphoma staging. It has excellent agreement with FDG-PET/CT and is a great alternative for managing lymphoma patients, without using ionizing radiation or an intravenous contrast agent.

## RESUMO

**CONTEXTO E OBJETIVO:**

A tomografia por emissão de pósitrons com 2-[18F]-fluoro-2-deoxi-D-glicose (FDG-PET/CT) tem sido defendida como método de escolha para o estadiamento do linfoma por realizar o estudo do corpo inteiro com boa sensibilidade para detecção das áreas acometidas e por combinar as capacidades de avaliação anatômica e funcional. Com os avanços tecnológicos, a ressonância magnética tem se apresentando como alternativa à FDG-PET/CT. Esta revisão sistemática com metanálise visa comparar a ressonância magnética de corpo inteiro (WB-MRI) com difusão com a FDG-PET/CT no estadiamento do linfoma.

**TIPO DE ESTUDO E LOCAL:**

Revisão sistemática de estudos de acurácia diagnóstica conduzida em universidade pública.

**MÉTODOS:**

Foi conduzida uma busca nos bancos de dados Medline, Embase, Scopus e Lilacs por estudos publicados até setembro de 2013 comparando a WB-MRI com a FDG-PET/CT no estadiamento do linfoma. As referências bibliográficas dos estudos incluídos foram checadas com a finalidade de encontrar citações adicionais relevantes.

**RESULTADOS:**

Foram incluídos seis estudos que avaliaram o estadiamento inicial do linfoma de 116 pacientes. A WB-MRI e a FDG-PET/CT concordaram em 90,5% dos casos (κ = 0,871; P < 0,0001). Na maioria dos estudos, quando houve discordância, a WB-MRI estabeleceu estadiamento superior à FDG-PET/CT. A sensibilidade da WB-MRI e da FDG-PET/CT, em relação ao padrão clínico-radiológico, variou de 59% a 100% e de 63% a 100%, respectivamente.

**CONCLUSÃO:**

A WB-MRI apresenta alta sensibilidade no estadiamento inicial do linfoma, excelente concordância com a FDG-PET/CT e representa uma ótima alternativa no manejo de pacientes com linfoma, sem utilizar radiação ionizante ou meio de contraste intravenoso.

## INTRODUCTION

Lymphomas account for approximately 5-6% of all malignancies.^1^ Over two-thirds of these cases are non-Hodgkin lymphomas (NHL), and Hodgkin’s lymphoma (HL) makes up the rest.^1^ After a histopathological diagnosis has been established, the imaging-based initial staging will influence the choice of therapy and prognosis, aid in radiation therapy planning for localized disease and provide a baseline for treatment response monitoring.^2^^,^^3^ HL and NHL staging is currently based on the Cotswolds modification of the Ann Arbor classification system.^4^ This system uses the number of tumor sites, the extent of involvement (nodal or extranodal) and its distribution as staging factors, whereas the Cotswolds modification also takes tumor burden into account.

Several imaging methods have been used for this purpose and, of these, computed tomography (CT) is currently the most popular.^2^^,^^3^ Over recent years, [18]F-fluoro-2-deoxyglucose positron emission tomography/computed tomography (FDG-PET/CT) has emerged as the most accurate method of all. It is based on the principle that malignant tissues exhibit higher glucose metabolism than that of healthy tissue^5^ and enables whole-body scanning with high sensitivity for detection of affected areas while combining the anatomical and functional assessment capabilities of CT and PET.^6^^,^^7^ However, its sensitivity and specificity vary according to histological subtype,^8^^,^^9^ and use of PET/CT has been correlated with substantial radiation exposure, particularly because scans must often be obtained repeatedly over the treatment course. Recent studies have shown that radiation exposure secondary to diagnostic imaging leads to increased lifetime risk of malignant tumors, especially in children.^10^^,^^11^^,^^12^

Magnetic resonance imaging (MRI) has emerged as a safer alternative for lymphoma staging, since progress in MRI techniques now enables rapid whole-body scanning^13^ while potentially providing the same information as FDG-PET/CT.^14^^,^^15^ The functional assessment in whole-body MRI (WB-MRI) is based on diffusion-weighted imaging (DWI), a method that maps water molecule movement in tissue (within cells, in the extracellular medium and across cell membranes). In the presence of lymphomas, the Brownian motion of water molecules is restricted due to increased tissue cellularity and elevated nucleus-to-cytoplasm ratio, which will produce relatively high signal intensity on DWI, compared with normal tissues.^16^ Using this principle, diffusion MRI can detect tumor-related changes that are not limited to anatomical information.^17^ Furthermore, apparent diffusion coefficient (ADC) quantification on DWI can provide useful information on treatment response and help distinguish benign from malignant lymph nodes.^18^

Over the last decade, a growing number of studies have compared WB-MRI and FDG-PET/CT in patients with lymphoma, using a variety of approaches. In studies focusing solely on initial lymphoma staging, the two methods are usually compared in two ways: taking into account the accuracy of each method for detection of individual lesions (on the basis of the number of lesions detected); or taking into account the final staging score, regardless of the number of lesions detected through each method.

Comparative analysis on these studies can be quite challenging when this attempts to focus on the ability of each method to detect individual lesions. The difficulty is mostly due to the wide range of WB-MRI protocols used, which precludes proper comparison. However, since the ultimate objective of initial lymphoma imaging is to define the disease stage at baseline, studies can be compared on the basis of the staging scores indicated by each method, regardless of the number of lesions detected.

## OBJECTIVES

Within this context, this study aimed to compare whole-body diffusion-weighted MRI (WB-MRI) with PET/CT for lymphoma staging by means of a meta-analysis, in order to identify whether the data available in the literature are sufficient to establish that WB-MRI is a safe alternative for lymphoma staging.

## METHODS

### Type of study and participants

This was a systematic review of diagnostic test accuracy studies, with meta-analysis. The spectrum of patients included HL and NHL cases.

The present study was approved by the local Research Ethics Committee, under number 0135/12HE.

### Inclusion criteria

All diagnostic test accuracy studies, comparing WB-MRI versus FDG-PET/CT for initial lymphoma staging, with the added utility of DWI in WB-MRI, which were published up to September 2013, were assessed.

### Exclusion criteria

Studies meeting any of the following criteria were excluded: data could only be extracted for one of the methods under analysis; FDG-PET/CT was used as the single reference standard for lymphoma staging; samples included cases previously reported elsewhere; the data represented a subpopulation analysis from larger investigations previously included in our review; the study included patients with diseases other than lymphoma; or the study assessed the performance of WB-MRI in relation to lymphomas, but only for detection of bone involvement.

### Search strategy

The Medline (via PubMed), Embase, Lilacs and Scopus databases were searched for relevant studies on the performance of WB-MRI versus other imaging methods for lymphoma evaluation. The references of each study included were checked for potentially relevant additional citations. The results from our search strategy are shown in Table 1. The search was last updated on September 27, 2013.

**Table 1.**
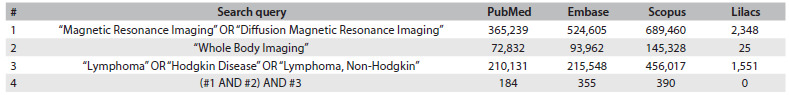
Search strategy results

Note: all synonyms under the hierarchical tree of each term were also included for each database.

### Article selection and quality assessment

For the first stage of the selection, two investigators (RR, AP) conducted independent assessments of the titles and abstracts of articles identified by the abovementioned search strategy. Studies on the diagnostic performance of WB-MRI for lymphoma staging or follow-up were included. Animal studies, reviews, meta-analyses, abstracts, editorials, letters to the editor, case reports, tutorials and practice guidelines were excluded. All clearly ineligible articles were also excluded.

For the second stage, all potentially eligible studies were set aside for full-text reading, critical appraisal and data extraction, conducted independently by the same investigators (RR, AP). Any disagreements arising between them at either stage were resolved through discussion and reaching a consensus.

Study quality was assessed using the Quality Assessment of Diagnostic Accuracy Studies (QUADAS-2).^19^ The QUADAS-2 tool enables more transparent ratings for bias and for the applicability of diagnostic accuracy studies. Three responses to questions regarding the risk of bias and applicability concerns were possible: “low”, “high” or “unclear”.

### Reference standard

Lymphoma staging provided by a set of clinical and radiological data was used to compare each technique in accordance with the Ann Arbor staging system. The final staging established needed to take into account all the clinical information available at the time of diagnosis, such as physical examination, laboratory and histological results, and bone marrow biopsy, and also the information available during the clinical and imaging follow-up (CT, FDG-PET/CT, WB-MRI or other methods). This follow-up was used to determine the status of lesions after treatment. For example, if they became larger during the follow-up period or decreased in size after treatment, they were considered positive for the presence of lymphoma.

### Statistical analysis

The Review Manager (RevMan) version 5.1 (Cochrane Collaboration, Oxford, England) was used to calculate sensitivity and specificity, with 95% confidence intervals (CIs), for WB-MRI and FDG-PET/CT, in comparison with a clinical-radiological standard reference. The results from each individual study were presented in forest plots.

Statistical analyses were performed using the SPSS 16.0 software package. The unweighted kappa (κ) statistic was used to test agreement between WB-MRI and FDG-PET/CT in the initial lymphoma staging. Agreement was considered poor at a κ value of 0, weak at 0.01-0.20, fair at 0.21-0.40, moderate at 0.41-0.60, good at 0.61-0.80 and excellent at 0.81-1.0.^20^ P values < 0.05 were considered indicative of significant differences.

## RESULTS

The search strategy chosen yielded 929 citations. After careful reading of the titles and abstracts and exclusion of duplicates, 19 articles were selected for full-text analysis and critical appraisal. Thirteen failed to meet the inclusion criteria (or met the exclusion criteria) and were excluded from further analysis: one study compared WB-MRI versus FDG-PET/CT for assessment of treatment response across a patient spectrum previously used in another study included in this systematic review;^21^ one study used FDG-PET/CT as a single reference standard for lymphoma staging;^22^ two studies assessed the performance of WB-MRI in lymphoma, but only for detection of bone involvement;^23^^,^^24^ two studies only compared WB-MRI with conventional CT;^25^^,^^26^ four studies had samples that included patients with diseases other than lymphoma;^27^^,^^28^^,^^29^^,^^30^ one study only compared WB-MRI with a reference standard;^31^ and two studies only compared WB-MRI with conventional CT and bone scintigraphy.^32^^,^^33^

On completion of the search and retrieval strategy, six prospective cohort studies were included for meta-analysis.^14^^,^^15^^,^^16^^,^^34^^,^^35^^,^^36^ Most of them either failed to conduct separate analyses on HL and NHL or conducted pooled analyses on different histological subtypes of NHL. The study by Wu et al.^34^ limited its analysis only to a single histological subtype of NHL. Table 2 provides a summary of the key features of these studies.

**Table 2.**
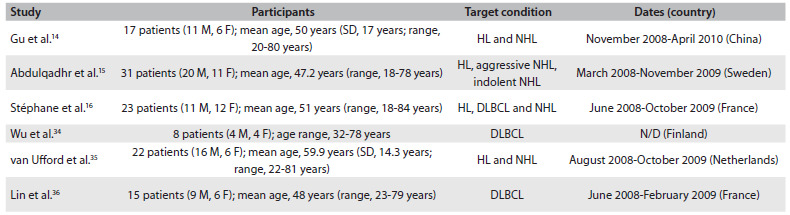
Key features of the studies included in this meta-analysis (all of them were prospective cohort studies)

M = male; F = female; SD = standard deviation; HL = Hodgkin’s lymphoma; NHL = non-Hodgkin lymphoma; DLBCL = diffuse large B-cell lymphoma; N/D = no data.

### Quality assessment on studies included

**Figure 1.**
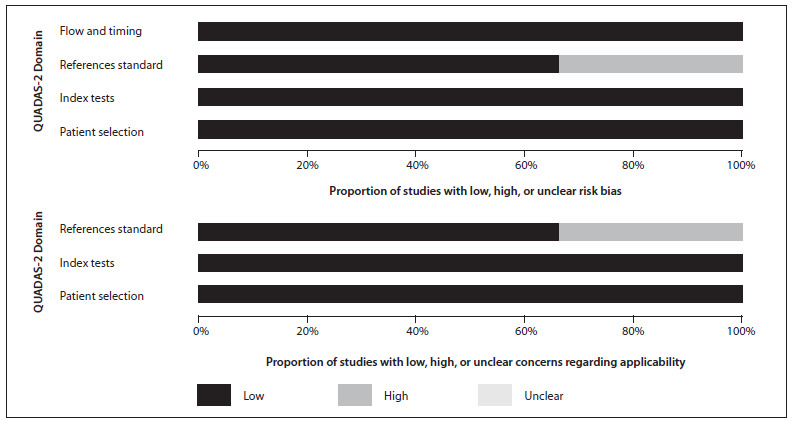
Graphical representation of study quality assessment.

Figure 1 summarizes the risk of bias and applicability judgments on the six studies included. The methodological quality graph presents the percentage of included studies for which the item was rated “low”, “high” or “unclear”, for each quality assessment domain. The graph shows that the potential area of concern was the description of the reference standard.

Patient selection criteria were clearly described in all the studies included. Regarding the reference standard, van Ufford et al.^35^ and Lin et al.^36^ did not describe it clearly. All the other quality assessment parameters were considered satisfactory across all six studies.

The QUADAS-2 score, expressed as a percentage of the maximum score, was 90% on average (range, 71-100%) in the six studies included. In the quality assessment, all of the studies were considered to present low risk of bias and low concerns about applicability.

### Summary assessment of the sensitivity of WB-MRI for lymphoma staging

**Figure 2.**
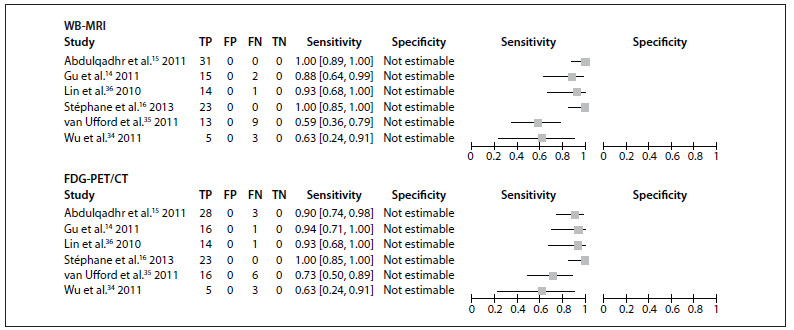
Forest plots of the sensitivity of imaging methods for lymphoma staging versus a comparison reference standard. TP = true positive, FP = false positive, FN = false negative, TN = true negative. Brackets show 95% confidence intervals. The figure shows the sensitivity for each study (squares) and 95% confidence intervals (horizontal lines). Specificity was not calculable, since all patients had lymphoma.

The sensitivity of WB-MRI and FDG-PET/CT for initial lymphoma staging versus that of the reference standard ranged from 59% to 100% and from 63% to 100% respectively (Figure 2). Gu et al.,^14^ Abdulqadhr et al.,^15^ Stéphane et al.^16^ and Lin et al.^36^ reported high sensitivity for both methods, whereas Wu et al.^34^ and van Ufford et al.^35^ found lower sensitivity values.

### Agreement between WB-MRI and FDG-PET/CT for lymphoma staging

In the study by Gu et al.,^14^ there was agreement between WB-MRI and FDG-PET/CT staging in 15 of their 17 patients. In the remaining two patients, WB-MRI overstaged one and understaged the other. In the latter patient, the staging with both methods was considered inadequate in relation to the reference standard because both of them failed to detect bone marrow infiltration, which was later confirmed by means of bone marrow biopsy. In the study by Abdulqadhr et al.,^15^ there was agreement between WB-MRI and FDG-PET/CT staging in 28 of their 31 patients. In the remaining three patients, low-grade lymphoma had higher staging through WB-MRI than through FDG-PET/CT, which was later validated by means of clinical staging. In the studies by Stéphane et al.^16^ and Wu et al.^34^ WB-MRI and FDG-PET/CT yielded the same staging in all patients, although three were incorrectly staged with both methods in the study by Wu et al.^34^ In the sample of van Ufford et al.,^35^ WB-MRI and FDG-PET/CT agreed regarding the staging of 17 of their 22 patients. WB-MRI overstaged five patients in relation to FDG-PET/CT, and only one of these patients, who had bone marrow infiltration later confirmed by biopsy, was correctly staged by means of the imaging method. Finally, in the study by Lin et al.,^36^ WB-MRI and FDG-PET/CT yielded similar staging for 14 patients. In the sole case in which the staging was different between the methods, it was higher with WB-MRI than with FDG-PET/CT, although both methods staged the patient incorrectly, compared with the reference standard. The kappa statistic was indicative of excellent overall agreement between WB-MRI and FDG-PET/CT (κ = 0.871 [0.782; 0.960]; P < 0.0001). Table 3 summarizes the agreement between the two methods in each study.

**Table 3.**
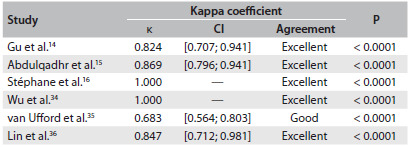
Agreement between WB-MRI and FDG-PET/CT for lymphoma staging in each of the studies included

WB-MRI = whole-body diffusion-weighted magnetic resonance imaging; FDG-PET/CT = [18]F-fluoro-2-deoxyglucose positron emission tomography; CI = confidence interval.

## DISCUSSION

The studies compared WB-MRI and FDG-PET/CT in terms of their ability to detect sites of disease involvement (both nodal and extranodal). This type of analysis is based on detection of individual lesions, i.e. each lesion regarded as positive counts toward the analysis on the agreement between the methods. Lesion-by-lesion comparison hinders meta-analysis on these studies, particularly because different methods are used in different studies. Differences in WB-MRI protocols, which may use distinct sequences with different acquisition planes, slice thicknesses and body areas (Table 4) are a particular cause for concern. Lymph node size cutoffs and the criteria used to classify an organ or extranodal lesion as “involved” also differed across studies. In view of this heterogeneity, lesions that were considered positive with one imaging method may have been classified as negative with the other.

**Table 4.**
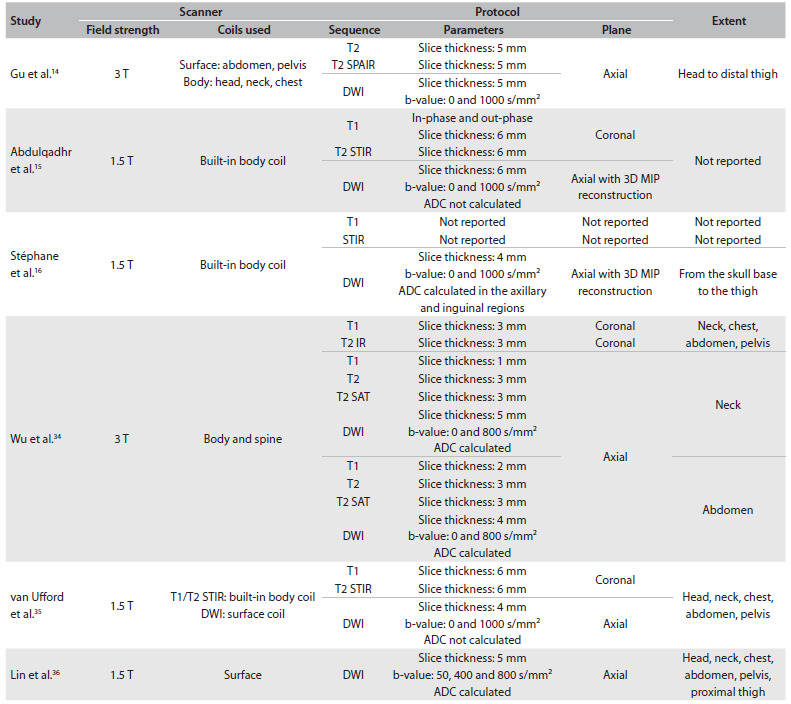
WB-MRI protocols used in studies included in the meta-analysis

SPAIR = spectral presaturation attenuated inversion recovery; STIR = short tau inversion recovery; DWI = diffusion-weighted imaging; SAT = fat saturation; ADC = apparent diffusion coefficient; MIP = maximum intensity projection; WB-MRI = whole-body diffusion-weighted magnetic resonance imaging.

However, all the studies also conducted staging in accordance with the Ann Arbor classification, which is based on a clinical and radiological reference standard, to which both WB-MRI and FDG-PET/CT staging were compared. Lymphoma staging in the Ann Arbor system is dependent on disease distribution (above or below the diaphragm) and on the affected lymph node sites. Detection of one abnormal lymph node or extranodal lesion suffices for a region to be classified as involved. Hence, for staging purposes, detection of additional lesions in a region or organ classified as positive is of no utility. This enables comparison between studies, despite methodological differences, in terms of the ability of each imaging method to establish staging.

Overall, the six studies included in this meta-analysis assessed 116 patients. There was agreement in staging between WB-MRI and FDG-PET/CT in 105 cases (90.5%). In nearly all cases of differences in staging, WB-MRI yielded a higher grade than FDG-PET/CT. Overall, there was excellent agreement between the two methods (κ = 0.871; P < 0.0001).

Some characteristics inherent to WB-MRI may lead to false-positive results, such as its limited ability to distinguish malignant from benign causes of lymph node enlargement, particularly in inguinal and axillary nodes, and its extreme sensitivity for small lymph nodes, even on DWI sequences,^14^ as well as the T2 shine-through effect, which refers to an area of high signal on DWI mimicking restricted diffusion due to very prolonged spin-spin relaxation time.^34^ In such cases, ADC quantitative analysis can be helpful for reducing the number of false positives in these cases. The causes of false-negative findings inherent to WB-MRI include diaphragmatic motion artifacts^37^^,^^38^ and artifacts in the hilar region due to respiratory and cardiac motion,^35^ as well as falsely elevated ADC values in these areas.^25^ Stéphane et al.^16^ also reported difficulties in analyzing hilar regions.

Several intrinsic factors may hinder interpretation of FDG-PET/CT results. Non-pathological variability in FDG uptake by healthy tissues, FDG uptake attributable to inflammation, altered biodistribution of FDG due to hyperglycemia or hyperinsulinemia and, particularly, the bone marrow activation commonly found in cancer patients after treatment may lead to false positives.^34^

Addition of DWI to WB-MRI protocols provides improved lymph node viewing, compared with conventional sequences, thus increasing the accuracy of the method for detection of lesions,^14^ whereas ADC value analysis improves specificity. In the study by Lin et al.,^36^ DWI using the lesion size criterion yielded sensitivity and specificity of 90% and 94% respectively, in comparison with FDG-PET/CT. Addition of visual ADC analysis reduced the sensitivity to 81% and increased the specificity to approximately 100%.

ADC quantification can also provide useful information on treatment response.^18^ In the study by Lin et al.,^36^ the mean ADC (×10^−3^ mm^2^/s) in regions with restricted diffusion was 0.75, versus 1.6 in regions with no restriction. In the study by Wu et al.,^34^ the ADC correlated inversely with the maximum standardized uptake value (SUV_max_) of FDG-PET/CT, thus suggesting that these parameters were comparable.

A different WB-MRI protocol was used in each of the six studies included, and this lack of standardization hindered comparison of WB-MRI with other, better established methods. Therefore, development of standardized protocols is critical to establishing the role of WB-MRI for staging and monitoring of lymphoma and other malignant conditions. Moreover, it may be of interest to compare different strength fields, such as 1.5 T versus 3.0 T. Despite this heterogeneity, five of the six studies included showed excellent agreement between WB-MRI and FDG-PET/CT, and one of them showed good agreement.^35^ Abdulqadhr et al.^15^ obtained T1-weighted and T2-weighted coronal images with SPAIR and axial DWI images, with 3D MIP reconstruction with a total scan time of roughly 50 minutes. Stéphane et al.^16^ used a similar protocol, except for the use of T2-weighted images with STIR, acquired for a total scan time of 40 minutes. Gu et al.^14^ used axial T2 and T2 with SPAIR sequences and axial DWI with 3D reconstruction for a total scan time of 44 to 52 minutes. Lin et al.^36^ also obtained excellent results with axial DWI alone and a total scan time of 30 to 45 minutes. This study also included ADC calculation, thus improving the specificity of the method, which is essential for treatment response assessment. Wu et al.^34^ also measured ADC and obtained results that corroborate its importance in patient follow-up, but the test protocol was complex and no information on total scan time was provided. van Ufford et al.^35^ used a protocol consisting of coronal T1-weighted, T2-weighted and STIR images (total scan time, 25 to 30 minutes) and axial DWI (total scan time, 20 to 25 minutes). This was the only study in which WB-MRI showed good agreement with FDG-PET/CT, and this was due to lack of experience in WB-MRI interpretation by the examining radiologists. In our opinion, a WB-MRI protocol can be built only with DWI, which has shown excellent results for lymphoma staging in comparison with FDG-PET/CT. ADC analysis, visual or otherwise, should also be provided for, since assessment of the functional evolution of residual lesions plays an important role in treatment monitoring.^39^

Diagnostic accuracy studies usually assess the accuracy of a test method under evaluation (index test) in relation to that of a gold-standard, well-established comparison method (reference standard), for detection of the presence or absence of a target condition. Conversely, the present review did not set out to assess the ability of WB-MRI or FDG-PET/CT to detect the presence or absence of lymphoma, but the ability of either method to yield a correct disease stage, in comparison with a reference standard. The clinical and radiological reference standard to which the results of WB-MRI and FDG-PET/CT were independently compared was based on a set of parameters assessed over time. Since this reference standard establishes the definitive baseline staging that will be used for patient management and treatment planning, it may be considered to be the true measurement. Therefore, we were able to calculate the sensitivity of the index methods as used in each of the studies included and compare them with the reference standard, i.e. to ascertain the ability of each method to stage the target condition correctly in relation to a true measurement.^40^ We found that both WB-MRI and FDG-PET/CT exhibited high sensitivity in the studies by Gu et al.,^14^ Abdulqadhr et al.^15^ and Lin et al.,^36^ ranging from 88 to 100% for WB-MRI and 90 to 94% for FDG-PET/CT. The highest sensitivity (100% for both methods) was found in the study by Stéphane et al.^16^ In the study by Wu et al.,^34^ because of a poorly representative patient spectrum and because both methods staged three out of the eight patients incorrectly, the overall sensitivity was 63%. In the study by van Ufford et al.,^35^ the sensitivity of WB-MRI was 59%, and that of FDG-PET/CT, 73%.

One limitation of the present review derives from the use of a clinical and radiological reference standard. Stéphane et al.^16^ used FDG-PET/CT as the gold standard method, although they also explicitly used clinical and imaging follow-up data to set up the differences between WB-MRI and FDG-PET/CT. Gu et al.^14^ also referred FDG-PET/CT as the reference standard for assessment of lesions on an individual basis and established the Ann Arbor staging using data such as physical examination, integrated FDG-PET/CT images at baseline and follow-up, and bone marrow biopsy results. Both Abdulqadhr et al.^15^ and van Ufford et al.^35^ separately staged the patients using WB-MRI and FDG-PET/CT. For the former, differences in staging between the two methods were resolved using biopsy results and clinical and CT follow-ups; for the latter, these differences were resolved using the contrast-enhanced full-dose component of the FDG-PET/CT examination and bone marrow biopsy. Lin et al.^36^ staged patients by means of physical examination, contrast-enhanced CT, FDG-PET/CT and bone marrow biopsy. Neither van Ufford et al.^35^ nor Lin et al.^36^ made it clear whether follow-up examinations were also included in determining the final staging. Wu et al.^34^ established lymphoma staging through detailed medical history, physical examination, standard laboratory tests, CT scans of the chest, abdomen and pelvis and bone marrow biopsy. All authors except Wu et al.^34^ included WB-MRI and/or FDG-PET/CT as part of the reference standard and, because of this, incorporation bias may have occurred, which would probably increase the level of agreement between the two index tests and the reference standard, and hence overestimate the measurements of diagnostic accuracy.^19^

Proven presence or absence of viable tumor tissue in anatomical pathology specimens is the most accurate reference standard in the field of oncology. However, since lymphomas often present as a diffuse disease, surgical exploration of all potential sites of involvement for histological analysis is ethically and practically unfeasible and may not affect treatment planning; therefore clinical and radiological staging is widely accepted as the reference standard.

WB-MRI provides several advantages over FDG-PET/CT. It does not emit ionizing radiation, which is particularly useful in children and young adults^41^^,^^42^ and when patients must undergo repeated imaging for follow-ups, as in lymphoma cases. FDG-PET/CT exposes patients to substantial radiation doses and, consequently, is associated with increased risk of later malignancies.^42^ Furthermore, thorough patient preparation is required before FDG-PET/CT, and because a cyclotron is required to produce FDG, it is not widely available.^43^

## CONCLUSION

WB-MRI is a highly sensitive method for initial lymphoma staging. It has excellent agreement with FDG-PET/CT and is a great alternative for managing lymphoma patients, without using ionizing radiation or an intravenous contrast agent. However, in order to define the role of WB-MRI in clinical practice, further studies are needed to assess the performance of WB-MRI in comparison with FDG-PET/CT, with regard to early and late response evaluation.
